# Templates of Lymph Node Dissection for Renal Cell Carcinoma: A Systematic Review of the Literature

**DOI:** 10.3389/fsurg.2018.00076

**Published:** 2018-12-19

**Authors:** Riccardo Campi, Francesco Sessa, Fabrizio Di Maida, Isabella Greco, Andrea Mari, Tána Takáčová, Andrea Cocci, Riccardo Fantechi, Alberto Lapini, Sergio Serni, Marco Carini, Andrea Minervini

**Affiliations:** ^1^Department of Urology, Careggi Hospital, University of Florence, Florence, Italy; ^2^Department of Experimental and Clinical Medicine, University of Florence, Florence, Italy; ^3^Department of Internal Medicine IV, Rheumatology, Clinical Immunology, Nephrology, HELIOS Dr. Horst-Schmidt-Kliniken Wiesbaden, Wiesbaden, Germany

**Keywords:** landmarks, lymphadenectomy, lymph node dissection, renal cell carcinoma, templates

## Abstract

**Background:** The role of lymph node dissection (LND) for renal cell carcinoma (RCC) is controversial. Notably, the conflicting evidence on the benefits and harms of LND is inherently linked to the lack of consensus on both anatomic templates and extent of lymphadenectomy. Herein, we provide a detailed overview of the most commonly dissected templates of LND for RCC, focusing on key anatomic landmarks and patterns of lymphatic drainage.

**Methods:** A systematic review of the English-language literature was performed without time filters in July 2018 in accordance to the Preferred Reporting Items for Systematic Reviews and Meta-analyses (PRISMA) statement recommendations. The primary endpoint was to summarize the most commonly dissected templates of LND according to the side of RCC.

**Results:** Overall, 25 studies were selected for qualitative analysis. Of these, most were retrospective. The LND template was heterogeneous across studies. Indications and extent of LND were either not reported or not standardized in most series. The most commonly dissected template for right-sided tumors included hilar, paracaval, and precaval nodes, with few authors extending the dissection to the inter-aortocaval, retrocaval, common iliac or pre/paraaortic nodes. Similarly, the most commonly dissected template for left-sided tumors encompassed the renal hilar, preaortic and paraaortic nodes, with few authors reporting a systematic dissection of inter-aortocaval, retro-aortic, common iliac, or para-caval nodes.

**Conclusions:** In light of the unpredictable renal lymphatic anatomy and the evidence from available prospective mapping studies, the extent of the most commonly dissected templates might be insufficient to catch the overall anatomic pattern of lymphatic drainage from RCC.

## Introduction

The role of lymph node dissection (LND) for renal cell carcinoma (RCC) is controversial (1–6). Indeed, while the latest Guidelines of the European Association of Urology (EAU) recommend considering an extended LND in patients with adverse clinical features and in the presence of clinically positive lymph nodes (LN) (5), the American Urological Association (AUA) and National Comprehensive Cancer Network (NCCN) Guidelines stressed that LND should be performed primarily for *staging* and *prognostic* purposes, and recommended not to perform *routine* LND in patients with clinically negative nodes (4, 7).

The lack of consensus on indications and value of LND stems from conflicting evidence on its ultimate impact on oncologic outcomes in both non-metastatic and metastatic patients with RCC (8–11). Yet, several factors may play a role in determining the outcomes of this procedure, such as surgical techniques, anatomic dissection templates and patient selection. In addition, there is lack of definitive knowledge on the anatomy of lymphatic drainage from RCC, which is unpredictable due to a wide heterogeneity of lymphatic vessels anatomy (12–14), the potential early hematogenous dissemination without LN infiltration (11, 12, 15) and the effects of local tumor progression (1).

Notably, as in other urological malignancies (16), the evidence on the anatomic templates of LND at the time of conservative or radical surgery for RCC is sparse and fragmentary (1, 2), making the interpretation of its benefits and harms challenging.

Therefore, we aimed to provide a detailed overview of the most commonly dissected templates of LND for RCC, focusing on key anatomic landmarks and patterns of lymphatic drainage.

## Methods

### Search Strategy

A systematic review of the English-language literature was performed without time filters using the MEDLINE (via PubMed), Cochrane Central Register of Controlled Trials and Web of Science (WoS) databases in July 2018 using the keywords ‘*lymph node dissection’ or ‘lymphadenectomy’ or ‘lymph nodes’ or ‘lymphatic drainage’ or ‘sentinel node’ or ‘mapping’* combined with ‘*renal cancer’* or ‘*renal cell carcinoma’* and ‘*template’* or ‘*landmark’* or ‘*extent’*. The review process was performed in accordance to the Preferred Reporting Items for Systematic Reviews and Meta-analyses (PRISMA) statement recommendations (17). A specific search strategy was designed using both free text and Mesh Terms. Hand-search of bibliographies of included studies and previous reviews on the topic was also performed to include additional relevant studies. Two reviewers (RC and FS) carried out the literature search independently.

### Inclusion Criteria

A specific population (P), intervention (I), comparator (C), outcome (O), and study design (S) (PICOS) framework was specified to define study eligibility, as recommended (17). In particular, the following criteria were outlined:

- Population (P): patients with non-metastatic or metastatic RCC;
- Intervention (I): conservative (partial nephrectomy) or radical (radical nephrectomy) surgery with LND;
- Comparison (C): patients undergoing surgery without LND (this criterion was not mandatory for inclusion of the studies in this review);
- Outcomes (O): anatomic extent of LND with detailed reporting of side-specific templates, including upper/lower boundaries of dissection, as well as of the number of patients undergoing LND;
- Study design (S): randomized-controlled or prospective/retrospective cohort studies with or without comparison of different LND templates.

Studies with insufficient reporting of the PICOS criteria were excluded. The primary endpoint of this review was to summarize the most commonly dissected templates of LND according to the side of RCC.

### Systematic Review Process

Mendeley reference software (Mendeley Ltd, London, UK) was used to identify and remove duplicates among records identified. Overall, 4,205 articles were preliminarily identified by the literature search. After exclusion of duplicates and articles not related to the topic of this review (*n* = 2,671), two independent reviewers (RC, FS.) screened titles and abstracts of 1,534 records. An *a priori* developed screening form was created to guide study selection. Disagreement was solved by a third party (AM), who supervised the systematic review process. After exclusion of case reports, book chapters, editorials, conference abstracts, animal studies, pre-clinical studies, previous reviews, and articles not related to the primary endpoint of this review, 103 articles were assessed for eligibility. Finally, 25 studies fullfilling all inclusion criteria were selected for qualitative analysis. The flow-chart depicting the overall review process according to PRISMA is shown in Supplementary Figure 1.

### Data Extraction

Data were extracted independently by two authors (IG, TT) in a-priori developed data extraction form. This included all relevant information on the anatomic landmarks of LND and on all elements of the PICOS framework, including study design, patient population, RCC characteristics and pathologic stage, indication for LND, extent of LND, LND metrics (number of LNs removed and of positive LNs, if provided) and specific description of LND templates according to tumor side (Table 1). A narrative form was used for qualitative data synthesis.

**Table 1:** Characteristics of studies included in the review with focus on study design, intervention, tumor stage, and anatomical templates of lymph node dissection in patients with renal cell carcinoma (RCC).

***N***	Authors ^*^= mapping study	**N of patients included** **(N of patients undergoing LND) ** **Study period ** **Study design**	**Intervention**	**Tumor pathological** **staging**	**N of LNs removed / location of sentinel LNs** ***(% of pN+ disease with location of positive*** ***LNs, if provided)***	**LND templates for right** **sided tumors**	**LND templates for** **left-sided tumors**	**Notes on LND templates**
1	Siminovitch et al. (18)	241 (102) 1968–1978 Retrospective, Single Center	Open RN	NR	NR NR	Extended LND (*n* = 19): resection of all nodal tissue from the diaphragm to aortic bifurcation Or Regional LND (*n* = 70): resection of ipsilateral nodes from the renal pedicle to the inferior mesenteric artery Or Hilar or LN biopsy: incidental lymph node resection		The extent of LND varied with intraoperative findings and surgeon's preference
2	Giuliani et al. (19)	200 (200) 1970–1987 Retrospective, Single Center	Open RN	pT1N0M0V0: 25 pT2N0M0V0: 50 pT3N0M0V0:21 pT4N0M0V0:1 N+V0M0 :20 M+V0:29 V+N0M0:24	LN removed: 30–40 34.5 % Location of positive LNs: Hilar 7 Rt, 9 Lt precaval 6 preaortic 4 retrocaval 8 retroaortic 9 laterocaval 4 lateroaortic 9 interaortocaval Rt 12, Lt 3 others Rt 2, Lt3	Hilar + Laterocaval, retrocaval, precaval, interaortocaval, preaortic	Hilar + Lateroaortic, preaortic, retroaortic, interaortocaval, precaval	Extended LND
3	Herrlinger et al. (20)	511 (511) 1970–1986 Retrospective, Single Center	Open RN	pT1–pT2 N0 M0: 109 LND vs. 82 no LNDpT3a N0 M0: 65 LND vs. 34 no LNDpT3b N0 M0:90 LND vs. 56 no LNDpT1–pT3 N1–N3 M0:65 LND vs. 19 no LND	NR (16.4%)	Paracaval, precaval, retrocaval, and interaortocaval from diaphragm to the bifurcation	Pre aortal, paraaortal, retroaortal from diaphgram to the bifurcation	Indication for LND: 320 planned extended LND; 191 facultative (if no cN+ or limited LND for staging purposes)
4	Minervini et al. (21)	167 (59) 1990–1997 Retrospective, Single Center	Open RN	pT1 31 (LND vs. 75 no LND) pT2 (11 LND vs. 20 no LND) pT3 5 (LND vs. 8 no LND) pT4 2 (LND vs. 5 no LND)	NR 5%	Anterior, posterior and lateral sides of the ipsilateral great vessel, from the level of the renal pedicle to the inferior mesenteric artery		LND performed at surgeon's discretion
5	Terrone et al. (22)	725 (608) 1983–1999 Retrospective, Multicenter	Open RN	pT1: 227 pT2: 107 pT3: 247 pT4: 27	median 9 (range 1–43) 13.6% Organ-confined disease (pT1–T2): pN+ disease was 3.4% in pts with < 13 LNs removed vs. 10.5% in pts with >13 LNs removed Locally advanced disease (pT3–T4): pN+ disease was 19.7% in pts with < 13 LNs removed vs. 32.2% in pts with >13 LNs removed	Anatomic limits of LND: From the crus of the diaphragm to the bifurcation of the aorta, including the primary lymph centers of the corresponding kidney		LND performed at surgeon's discretion
6	Terrone et al. (23)	735 (618) 1983–1999 Retrospective Multicenter	Open RN	pT1aN0: 85 pT1aN+:0 pT1bN0:141 pT1bN+:7 pT2N0: 91 pT2N+: 13 pT3aN0: 104 pT3aN+: 2 pT3b/cN0: 101 pT3b/cN+: 27 pT4N0: 8 pT4bN+: 20	Median 13 (1-35) [pN+ pts]; 9 (1-43) [pN0 pts] Median 3 (1-18) [pN+ pts]; 0 (0–0) [pN0 pts] (14.2%)	From the crus of the diaphragm to hilar, precaval, retrocaval, laterocaval and interaortic	From the crus of the diaphragm to hilar, preaortic, retroaortic, lateroaortic	LND not performed or limited to renal hilum in pts with advanced age or significant comorbidity affecting life expectancy
7	Simmons et al. (24)	700 (14) 1997–2006 Retrospective, Single Center	Laparoscopic RN	NR	Median 1 (range 1–9) 57%	Limits of LND: from the diaphragmatic crus to the level of the aortic bifurcation. anterolateral to the aorta with the extention to the vena cava	Limits of LND: from the diaphragmatic crus to the level of the aortic bifurcation anterolateral to the aorta	LND performed in case of clinical lymphadenopathy
8	Chapman et al. (25)	100 (50) 2006–2007 Retrospective, Single Center	Laparoscopic RN	pT1: 27 pT2: 10 pT3: 12 pT4: 1	Left sided: mean 10,5 (range 0–25) Right sided: mean 4,8 (range 0–18) Extended LND Left sided: 14.3 (range 5–20) Right sided: 8.8 (range 4–14) Positive LNs: 10%; Paraaortic 3; Paracaval 1; Retrocaval 1	Limits of LND: adrenal vein, bifurcation of the common iliac vein, Gerota's fascia, and medial aspect of the vena cava	Limits of LND: from the crus of the diaphragm to the bifurcation of the common iliac artery, Gerota's fascia, and the medial aspect of the aorta	The last four LNDs included formal dissection of retrocaval nodes
9	Capitanio et al. (26)	3507 (3507) 1984–2001 Retrospective Multicenter	Open RN or PN	1145 T1a (1137 pN0, 8 pN1–2); 901 T1b (886 pN0, 15pN1–2); 468 T2 (448 pN0, 20pN1–2); 993 T3 (871 pN0, 122 pN1–2)	NR (4.7%)	Hilar + ipsilateral side of the great vessels		On the basis of surgeon preference, more extensive LND included inter-aorto-caval nodes
10	Blom et al. (15)	772 (362) 1988–1991 Prospective Phase III Multicenter RCT	Open RN +/– LND	pT0: 4 pT1: 21 pT2: 221 pT3:101 pT4: 3 pTX: 3	NR 14/346 (4%)	From the crus of the diaphragm inferiorly to the bifurcation of the aorta; lateral caval, precaval, postcaval and interaortocaval nodes	From the crus of the diaphragm inferiorly to the bifurcation of the aorta; left para-aortic nodes, the left diaphragmatic nodes, and the preaortic nodes	When previously undertected enlarged lymph nodes were found during operation in a patient in the nephrectomy-only treatment group, lymph-node biopsy, or sampling was done for staging purposes, but a complete lymph-node dissection was not performed
11	Ming et al. (27)	702 (114) 1997–2007 Retrospective, Single Center	Open RN	pT1: 29 pT2: 54 pT3: 29 pT4: 2 (in patients with enlarged LNs)	NR 36/114 (31.6%) 32/36 (89%) of pts with pN+ had positive frozen section analysis	Anatomic limits of LND: Anterior, posterior, and lateral sides of the ipsilateral great vessel, from the level of the renal pedicle to the inferior mesenteric artery		17/114 (15%) patients had distant metastates Frozen section examination (FSE) of *enlarged* LNs at the time of RN The sensitivity, specificity, concordance, and false-negative rate of FSE was 88.9%, 100%, 96.5%, and 11.1%, respectively.
12	Crispen et al. (28)	169 (169) 2002–2006 Retrospective, Single Center	Open RN	pT1: 4 pT2: 24 pT3: 133 pT4: 8	Overall: median 6 (IQR 3–13) In pts with pN+ disease: median 6.5 (IQR 2–15) 38% Right-sided tumors (*n* = 35): 43% hilar 57% pre-para caval 20% interaortocaval 20% pre-aortic 0% left hilar Left-sided tumors *(n* = 29) 76% hilar 62% pre-para aortic 14% interaortocaval 7% pre-paraacaval 0% right hilar	Hilar, Pre-para caval, interaortocaval, pre-aortic, left hilar	Hilar, pre-paraaortic, interaortocaval, para-caval, right hilar	Of the 64 pts with pN+ disease, 45% had no metastases in the peri-hilar region. No patient with right-sided tumor had positive LN metastasis in the para-aortic without metastases in other retroperitoneal LNs No patient with left-sided tumor had LN metastases in the para-caval LNs without involvement of para-aortic or interaortocaval LNs
13	Abaza et al. (29)	36 (36) 2008–2010 Prospective, Single Center	33 RRN 3 RAPN	pT1a: 8 pT1b: 6 pT2: 6 pT3a: 10 pT3b:6	For RRN: 13,7 (3-31) For RAPN: 15.3 (11-22) (3%)	Hilar + pericaval, retrocaval, interaortocaval	Hilar + preaortic, periaortic, interaortocaval	LND performed at surgeon's discretion
14	Bex et al. (30)^*^	20 (20) Prospective, Single Center	7 Open RN 9 Open PN 4 Lap RN	All pT1a–pT2, pN0 cM0	Sentinel LNs (*n* = 26): 17 interaortocaval 1 hilar 1 celiac trunk 1 internal mammary chain 1 mediastinal 1 pleural 4 retrocaval 8 paraaortic 5 not visualized (0%)	Hilar, paracaval,interaortocaval + SNs	Hilar, paraaortic, interaortocaval + SNs	Mapping study on the distribution of sentinel LNs
15	Delacroix et al. (31)	2521 (NR) 1995–2009 Retrospective, Single Center	Open RN	NR	mean 9 median 6 (2.7%)	“full bilateral” LND: from the crus of the diaphragm to the bifurcation of the aorta Or Ipsilateral great vessels or interaortocaval LNs Or Ipsilateral great vessels exclusively		Indications for LND: Clinically node positive disease or presence of at least 2 high risk features (size >10 cm, cT3, or greater disease, sarcomatoid feauters, GN3 or greater) The template of LND was not standardized
16	Kwon et al. (32)	1503 (763) 1990–2007 Retrospective, Single Center	Open RN or Open PN or Lap RN	pT1a (215 LND vs. 472 no LND); pT1b (244 LND vs. 168 no LND); pT2 (127 LND vs. 50 no LND), pT3–T4 (176 LND vs. 50 no LND)	5 (range 1–33) 2.5%	Lateral caval, precaval, postcaval, interoartocaval	Left paraortic, left diaphragmatic, preoartic	The most frequent site of positive LN was the hilar location
17	Capitanio et al. (33)	1847 (44) 1987–2011 Retrospetive, Single-Center	Open RN	All pT4 (*n* = 44)	mean 11.8 median 8 (range 1–37) 56.8% Mean 4.8 N+ mean 4.8 median 2 (range 0–36)	Regional: hilar plus precaval (systematically performed) Extendend: regional plus retrocaval from the adreanal vein to the aortic bifrucation and interaortocaval	Regional: hilar plus paraortic (from the adrenal vein to the aortic beforcation) (systematically performed) Extendend LND: regional plus preaortic (from the crus to aortic bifurcation) and interaortocaval	Extended LND performed at surgeon's discretion (48%) Of 28 pts with suspected lymphadenopathy, 89% had pN+ disease
18	Mehta et al. (34)	871 (333) 1992–2012 Retrospective, Single Center	Open or laparoscopic RN	pT1: 67 pT2: 63 pT3: 177 pT4: 26	Mean 8.3 (1-83) 26% Hilar: 45% Non-Hilar: 46% Both hilar + non-hilar: 9%	Hilar, paracaval/precaval, right common iliac, and interaortocaval	Hilar, para-aortic/ preaortic, left common iliac, interaortocaval	Suprahilar LNs were not routinely removed (considering from the area between the upper pole of the kidney and the ipsilateral great vessel above the level of the renal vein)
19	Capitanio et al. (10)	1983 (874) 1987–2011 Retrospective, Single-Center	712 PN 1271 RN (open or laparoscopic)	Overall (*n* = 1983) pT1: 1247 pT2: 220 pT3: 319 pT4: 42	Mean 7.8 Mean *n* of LNs removed according to LND template: Limited LND (45%): 3.1 Regional LND (34%): 9.7 Extended LND (21%): 14.8 Overall LN invasion (pts undergoing PN or RN, *n* = 874): 13.7% LN invasion in pts undergoing RN (*n* = 844): 14.2% LN invasion rates were 1.9, 8.3, 22.3, 54.3% in pT1,pT2,pT3, and pT4 disease, respectively.	Limited LND: ipsilateral hilar regional LNs Side specific LND: hilar region plus, on the right side, precaval nodes or paraortic nodes on the left side, from the adrenal vein to the level of the aortic/caval bifurcation. Extended: regional plus on the left paraortic and preaortic nodes and on the right retrocaval and precaval nodes to the aortic/caval bifurcation.		Interaortocaval (from the midline of the inferior vena cava to the midline of the aorta); precaval and retrocaval (from the midline of inferior vena cava to the right ureter) Extended LND: from the crus of the diaphragm to the aortic bifurcation
20	Feuerstein et al. (8)	258 (177) 1992–2013 Retrospective single center	Open CN	pT1M+: 11 pT2M+: 19 pT3M+: 147	NR (33%)	Hilar, paracaval, precaval, retrocaval and interaortocaval	Hilar paraaortic, preaortic and interaortocaval.	On the basis of surgeon preference, limited hilar LND was performed in 30 (17%) pts
21	Feuerstein et al. (35)	524 (334) 1990–2012 Retrospective Single Center	392 RN 32 PN (Open 301 minimal invasive surgery 33)	pT2: 95 pT3: 227 pT4: 12	NR (8%)	Hilar, paracaval, precaval, retrocaval and interaortocaval	Hilar, paraaortic, preaortic and interaortocaval.	Indications for LND: Clinically node positive disease, on the basis of surgeon preference; limited hilar LND was performed in 40 (20%) pts
22	Babaian et al. (36)	1270 (564) 1993–2012 Retrospective, Single Center	RN or PN (Approach NR)	pT any	pN0 group: median 5 (range 1–33) pN1 group: median 8 (range 1–42) cN1 pN0 : 37% cN0 pN1: 7.2% 23% Median n. of positive LNs: 2 (IQR 1–39)	Standard or extended retroperitoneal LND		LND template performed at surgeon's discretion
23	Kuusk et al. (37)^*^	68 (40) 2008–2017 Prospective phase II single Center trial	Open RN	pT1a: 6 pT1b. 21 pT2a: 6 pT2b: 2 pT3a: 5	Sentinel LNs: median 1 (IQR 1–2) (2.5%) Drainage from right-sided tumors: mainly inter-aortocaval, retrocaval Drainage from left-sided tumors: mainly paraaortic	Renal hilar, paracaval, retrocaval, precaval and interaortocaval LNs from the upper margin of the crus of the diaphragm down to the right common iliac artery crossing the inferior vena cava On the right side 6/18 pts had sentinel LNs had simultaneous drainage to interaortocaval, retrocaval, left preaortic or paraaortic and left supraclavicular LNs.	Renal hilar, paraaortic, retroaortic and preaortic LNs from the level of the crus to the bifurcation of the aorta Only 3 pts had direct left renal hilar sentinel LNs	Mapping study using lymphoscintigraphy + SPECT/CT scan 14 pts (35%) had SNs outside the respective locoregional retroperitoneal template, of whom 8 (20%) supradiaphragmatic SNs
24	Dell'Oglio et al. (38)	2010 (640) 1990–2014 Retrospective Single Center	Open PN or RN	NR	median 5 (IQR 3–8) 2.2% 2 precaval 4 retrocaval 2 interaortocaval 7 paraaortic	Paracaval, retrocaval, and precaval nodes from the adreanal vein to the level of the inferior mesenteric artery	Paraaortic and preaortic nodes from the crus of the diaphragm to the inferior mesenteric artery	Standardized LND templateInteraortal nodes were removed according to the clinical judgment of the surgeons in 13.6% of pts
25	Nini et al. (39)	2844 (451) 180–2012 Retrospective multicenter study	Open RN	pT1a: 26 pT1b: 74 pT2a: 43 pT2b: 12 pT3a: 100 pT3b: 105 pT3c: 28 pT4: 27	mean 15 median 14 (IQR 9–19) Positive LNs: (23%) Interaortocaval 26% hilar 20% side specific 54%	Hilar and side specific (pre and paracaval) and interoaortocaval nodal stations	Hilar and side specific (pre and para aortic) and interoaortocaval nodal stations	27% of patients were metastatic at diagnosisIndications for LND: on the basis of surgeon preference

*CN, cytoreductive nephrectomy; IQR, interquartile range; LND, lymph node dissection; LNs, lymph nodes; N, number; NR, not reported; PN, partial nephrectomy; pts, patients; RAPN, robot assisted partial nephrectomy; RCT, randomized controlled trial; RN, radical nephrectomy; RRN, robotic radical nephrectomy; SNs, sentinel nodes*.

* *Mapping studies evaluating the lymphatic drainage from renal tumors*.

## Results

### Characteristics of Included Study and Quality of Reporting LND Templates

The key characteristics and findings of the studies included in the review are shown in Table 1. Of the 25 studies included in final qualitative analysis, 21 were retrospective, either single- (*n* = 17) or multicenter (*n* = 4), while 3 were prospective single-center. One multicenter randomized-controlled trial (RCT) was included (15). Only two studies were specifically designed to evaluate the pattern of lymphatic drainage from RCC by using sequential lymphoscintigraphy and sentinel node (SN) biopsy (30, 37). No study compared LN yield, cancer control and surgical complications of *extended* vs. *limited* LND. Risk of bias assessment for the studies included in the review is depicted in Supplementary Table 1. Overall, the quality of evidence according to Grading of Recommendations Assessment, Development, and Evaluation (GRADE) is low.

Overall, the proportion of patients undergoing LND was heterogeneous across included studies and indications for LND were not standardized in most series, being based on surgeon's preference and/or according to intraoperative suspicion of LN metastases (Table 1). Most studies described an open surgical approach for both nephrectomy and LND. A minimally invasive approach was used in selected series, using either a laparoscopic (*n* = 7) (8, 10, 24, 25, 30, 34, 35) or robotic approach (*n* = 1) (29). Pathologic tumor stage was heterogeneous across included studies, although most series considered locally advanced tumors (pT stage 3–4) (15, 22, 23, 28, 34, 35, 39). One study examined the role of LND at the time of cytoreductive nephrectomy (8).

The overall number of LNs removed at the time of surgery (10, 19, 22–25, 28–34, 36–39), as well as the proportion of positive LNs, were reported by the majority of included studies, despite the latter was highly variable across the included studies (range 0–56,8%) (Table 1). Of note, only a minority of reports described the specific anatomic location (and number) of positive nodes (19, 25, 28, 30, 34, 37–39). All included studies reported a *side-*specific anatomical template of LND, with description of the anatomical boundaries and upper/lower limits of dissection; yet, indications and extent (i.e., standard vs. extended) of LND were either not reported or not standardized in most series. Furthermore, only few authors reported the lateral landmarks of dissection for the paracaval/paraaortic templates [i.e., the right and left ureter, respectively (10)].

**Figure 1:**
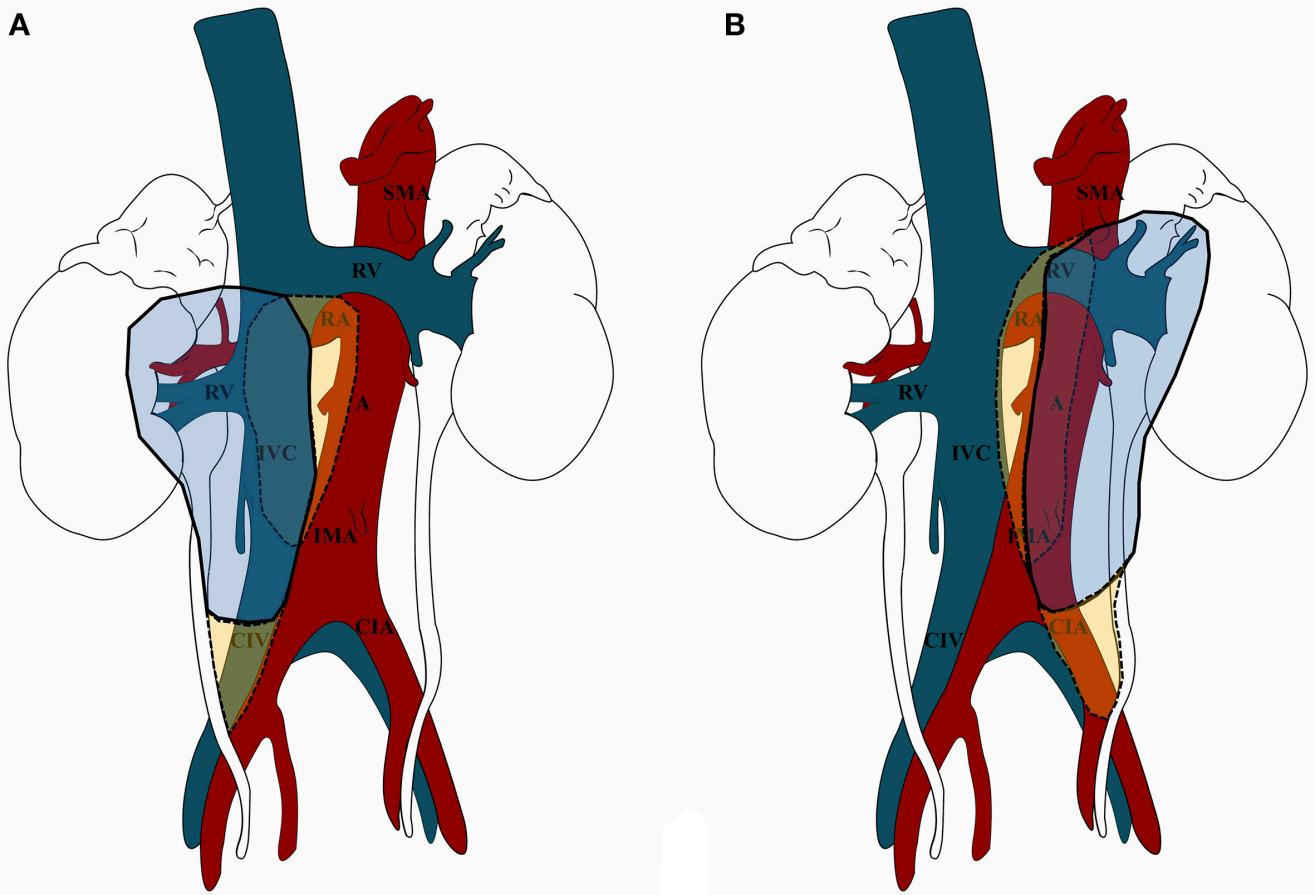
Overview of the most commonly dissected templates of lymph node dissection (LND) for renal cell carcinoma (RCC) according to tumor side (A. Right-sided tumors; B. Left-sided tumors). **(A)** For right-sided tumors, LND included in most cases (continuous line) the renal hilar, paracaval, and precaval nodes, from the crus of the diaphragm to the aortic bifurcation (*in blue*). Extended LND (dotted line) also included the inter-aortocaval/retrocaval nodes and the right common iliac nodes (*in yellow*). **(B)**. For left-sided tumors, LND included in most cases (continuous line) the renal hilar, preaortic, and paraaortic nodes, from the crus of the diaphragm to the aortic bifurcation (in *blue*). Extended LND (dotted line) also included the inter-aortocaval/retroaortic nodes and the left common iliac nodes (in *yellow*). A, aorta; CIA, common iliac artery; CIV, common iliac vein; IMA, inferior mesenteric artery; IVC, inferior vena cava; RA, renal artery; RV, renal vein; SMA, superior mesenteric artery.

The overview of the most commonly dissected templates of LND for RCC according to tumor side is shown in Figure 1, while a detailed analysis of the specific templates considered in each study included in the review in Table 1. Of note, it was not possible to define a putative pattern of lymphatic spread from RCC due to lack of information on the location of positive LNs within the anatomical sites of the template in most series.

### Templates of Lymph Node Dissection for Right-Sided RCC

For right-sided tumors, the LND template included in most cases the hilar, paracaval (on the lateral side of the vena cava), and precaval (i.e., on the anterior side of the vena cava) nodes, from the crus of the diaphragm to the aortic bifurcation (Table 1). Some authors reported the dissection of a more *extended* template including the inter-aortocaval (8, 15, 19, 20, 25, 28–30, 32–35, 37, 39), retrocaval (8, 15, 19–21, 23, 27, 29, 32, 35, 37, 38), common iliac (25) nodes or even pre/paraaortic nodes (19). In their retrospective study on the outcomes of laparoscopic radical nephrectomy with or without LND, Chapman et al. included the removal of inter-aortocaval and retrocaval nodes only in the last group of patients included in the series (25). Notably, Abaza et al. showed the feasibility of performing LND for right-sided tumors including paracaval, retrocaval, and inter-aortocaval nodes using a robotic approach (29).

The study by Nini et al. evaluated the pattern of nodal metastatic dissemination from RCC in a cohort of patients undergoing extended LND including removal of hilar, pre/paracaval and inter-aortocaval nodes (39). The authors found that, in case of right-sided tumors, the positive LNs were located in the paracaval, interaortocaval, and renal hilar regions in 44, 40, and 16% of cases, respectively. When two nodal areas were involved, they included the paracaval + interaortocaval regions in 87% of cases. It is important to highlight that in their retrospective cohort study, Crispen et al. reported that, despite the location of LN metastases was related to the tumor side, 45% of patients with pN+ disease had no metastases in the perihilar nodes and no patient with a right-sided tumor had involvement of the para-aortic LNs without metastases in other retroperitoneal lymph nodes (28).

### Templates of Lymph Node Dissection for Left-Sided RCC

For left-sided tumors, the most commonly dissected anatomical templates were the renal hilar, pre/para aortic (i.e., on the anterior and lateral side of the aorta) from the crus of the diaphragm to the aortic bifurcation (Table 1). Of note, only few authors reported a systematic dissection of a more *extended* template including inter-aortocaval (8, 19, 25, 28–30, 34, 35, 39), retro-aortic (19–21, 23, 27, 28, 37), common iliac (25), or even para-caval (28) nodes. A LND template including periaortic and interaortocaval nodes was also described by Abaza et al. in their study including patients undergoing robotic LND (29). In their study on extended LND, Nini et al. reported that positive LNs from left-sided tumors were located in the pre/paraaortic, interaortocaval, and renal hilar regions in 67, 9, and 24% of cases (39). When two nodal areas were involved, they included the pre/paraaortic + renal hilar regions in 91% of cases. Of note, in the retrospective study by Crispen et al. (28), no patient with a left-sided tumor had positive paracaval LNs without involvement of para-aortic or interaortocaval nodes.

### Mapping Studies Using the Sentinel Node Technique Assessing the Pattern of Lymphatic Drainage From RCC

Two studies included in the review were specifically designed to evaluate the pattern of lymphatic drainage from RCC using sequential lymphoscintigraphy and sentinel node biopsy (30, 37). In the largest prospective phase II single-arm study evaluating the distribution of sentinel LNs and the lymphatic drainage pattern of renal tumors *in vivo* with SPECT, Kuusk et al. found that drainage from right-sided tumors was predominantly into inter-aortocaval and retrocaval sentinel nodes (37). Moreover, 6/18 (33%) patients with right-sided tumors had simultaneous drainage to interaortocaval, retrocaval, left preaortic or para-aortic, and left supraclavicular lymph nodes. Only three patients had sentinel LNs in the right paracaval and renal hilar and no patient to precaval LNs. The distribution of sentinel LNs from left-sided tumors revealed that the lymphatic drainage was mainly into para-aortic LNs and that very few patients had direct left hilar sentinel LNs (37). Moreover, 9/22 (41%) had simultaneous renal hilar, mediastinal, left supraclavicular, retrocrural, left common iliac, renal fossa, and interaortocaval sentinel LNs.

## Discussion

Lymph node dissection has staging, prognostic and potentially therapeutic roles in several urologic malignancies, including prostate (40), bladder (41), testis (42), and upper tract urothelial (16) tumors. Of note, its role in the management of RCC is still debated (3–5, 7). Accordingly, a recent large European multi-institutional study reported a trend toward lower rates of LND over time for patients undergoing radical or partial nephrectomy for RCC (43). The most recent systematic review on this topic (3) concluded that, although LND yields independent prognostic information, the existing literature does not support a therapeutic benefit in either non-metastatic or metastatic RCC. Nonetheless, LND may have a role in selected high-risk non-metastatic patients, for whom further prospective studies are warranted (3). Indeed, the extent of LND has been shown to affect cancer-specific survival and metastatic progression in specific sub-categories of patients with RCC (10).

This conflicting evidence on the benefits and harms of LND for RCC (1, 9, 11, 44), which directly impacts the strengths of Guidelines recommendations (4, 5, 7), is inherently linked to the lack of consensus on both anatomic templates and extent of lymphadenectomy (4). In this regard, some authors advocate routine *extended* LND (if indicated) (6); yet, the potential oncologic benefits should be balanced with the increased risk of surgical complications, which may be not negligible in this setting (45). Beyond the lack of standardized reporting of surgical templates of LND in the literature (including the only RCT available to date (1, 2, 14, 15), the complex anatomy of lymphatic drainage from RCC undermines a thorough understanding of the anatomic sites of lymphatic involvement.

Therefore, in this review we provided a detailed assessment of the most commonly dissected templates of LND for RCC, focusing on the key anatomic landmarks and on the putative patterns of lymphatic drainage, as suggested by available mapping studies.

The available autopsy studies underscore the unpredictable variability of drainage patterns and the heterogeneity of lymphatic metastases from RCC; as such, they provided only limited evidence for defining effective LND templates to date (1, 12, 13, 46–48). Overall, four critical issues should be considered when evaluating potential anatomic templates of LND for RCC. First, the presence of *anterior, intravascular*, and *posterior* bundles of efferent lymphatic vessels from both the right and left kidney, whose drainage pattern is still not completely understood (12). These efferent lymphatics drain into paracaval, precaval, retrocaval, and interaortocaval nodes, for right-sided tumors, and into paraaortic, preaortic, and retroaortic nodes, for left-sided tumors (13, 46). Second, the existence of peripheral lymphovenous communication sites located in the inferior vena cava at the level of the renal veins or through the thoracic duct (12); these communications may imply LN station skipping and explain how distant metastases can occur without concurrent retroperitoneal LN involvement (14, 46). Third, the effects of local tumor growth may be responsible for further unpredictability (2). Finally, renal hilar regions have been shown to be rarely affected by LN metastases (49, 50).

The anatomy of lymphatic drainage from RCC has been also investigated *in vivo* taking advantage of sentinel LN biopsy (12, 30, 37, 51). Although this technique may be theoretically challenged by tumor lymphangiogenesis and lymphatic remodeling, the largest mapping study available to date showed that drainage from right-sided tumors was predominantly into inter-aortocaval and retrocaval sentinel nodes while drainage from left-sided tumors mainly into para-aortic LNs (37). Moreover, only a negligible proportion of patients had sentinel LNs in the ipsilateral renal hilar regions.

Our review provides relevant information to better contextualize the current evidence on anatomic templates of LND for RCC.

A key finding is that the surgical template for LND was heterogenous across the included studies and only a minority of reports described the specific anatomic location (and number) of positive nodes within the template (Table 1). Moreover, indications and extent of LND were either not reported or not standardized in most series and relied mainly on surgeon's judgement. An additional finding is that the most commonly dissected template for right-sided tumors included hilar, paracaval, and precaval nodes, with few authors extending the dissection to the inter-aortocaval, retrocaval, common iliac, or pre/paraaortic nodes (Table 1). As such, considering the findings of the available mapping studies using the SN technique (30, 37) as well as those of studies reporting an *extended* LND in high-risk patients (28, 39), the extent of the most commonly dissected templates for right-sided tumors (especially if advanced in stage) might be insufficient to catch the comprehensive pattern of lymphatic drainage, which frequently involves the retro-caval and inter-aorto-caval sites. Similarly, the most commonly dissected template for left-sided tumors encompassed the renal hilar, pre/para aortic nodes, with few authors reporting a systematic dissection of inter-aortocaval, retro-aortic, common iliac, or para-caval nodes (Figure 1). In conclusion, in light of the above discussed anatomic considerations, the templates of LND most commonly dissected for both right- and left-sided tumors might be unable to capture the complexity of the LN drainage from RCC. This concept is reflected in the relatively low number of LNs removed in most of the included studies (Table 1).

The findings from our review should be interpreted in light of several limitations at both a review- and study-level. Our review was not designed to assess the association of LND templates with positive LNs and key oncologic outcomes after surgery for RCC. As such, we were unable to evaluate the potential impact of extent of LND and cancer-specific or overall survival after surgery. Second, the review strategy might not have been able to identify all relevant studies on the topic of interest. In particular, a limitation of our review is that studies that did not fulfill the pre-specified eligibility criteria (including detailed reporting of anatomical LND templates) were excluded from the qualitative synthesis. Therefore, studies that attempted to address the role of *extended* vs. *limited* LND which did not adequately report the anatomic sites of LND were not included in our review. Third, most studies included in qualitative analysis were retrospective, subject to selection bias. Moreover, patient populations, surgical approaches, techniques for LND and quality of reporting LND results were highly heterogeneous across included studies, reducing the generalizability of findings. In this regard, due to the low number of series reporting LND during robotic or laparoscopic surgery, the above mentioned LND templates may not be entirely applicable to minimally-invasive surgery. Finally, it was not possible to evaluate the potential association between number of LNs removed (which has been associated with rate of LN metastases and cancer-specific survival (10, 11, 22) and anatomic extent of LND template, due to the lack of this information in most series.

Our review affords opportunities for significant further research in this field. In particular, future RCTs should evaluate the benefits and harms of *extended* LND using standardized anatomic templates in patients with high-risk RCC, where LND may significantly impact on the disease course (45). Such studies should aim to build an evidence-based consensus on the surgical management of retroperitoneal nodes in patients with RCC, overcoming the complexity of renal lymphatic anatomy. To this aim, an effective study design should compare *extended* vs. *no* LND to avoid risk of misclassification of pN status due to the confounding effect of a *limited* LND arm (52). Future mapping studies should improve *in vivo* evaluation of lymphatic drainage from RCC, taking advantage of sentinel node (37) and frozen section analysis (27), novel technologies (including indocyanine green fluorescent lymphography) and advanced multimodality imaging (12). Finally, further research should explore the role of LND during minimally-invasive surgery to assess the feasibility and outcomes of extended surgical templates.

## Conclusions

The conflicting evidence on the benefits and harms of LND for RCC is inherently linked to the lack of consensus on anatomic templates and extent of lymphadenectomy. In this review, we provided an overview of the most commonly dissected templates of LND, focusing on key anatomic landmarks and patterns of lymphatic drainage. The surgical template for LND was heterogenous across included studies, and indications and extent of LND were either not reported or not standardized in most series.

For right-sided tumors, the most commonly dissected templates were the hilar, paracaval, and precaval nodes, while for left-sided tumors, the renal hilar, pre/para aortic nodes, both from the crus of the diaphragm to the aortic bifurcation.

In light of the unpredictable renal lymphatic anatomy and considering the evidence from available prospective mapping studies, which suggest that lymphatic drainage from renal tumors may be directed predominantly to inter-aortocaval and retrocaval nodes—on the right side—and para-aortic and inter-aortocaval nodes—on the left side, the extent of the most commonly dissected templates might be insufficient to catch the overall anatomic pattern of lymphatic drainage from RCC, especially for higher stage and right-sided tumors.

## Author Contributions

RC and AMi contributed conception and design of the study. RC and FS performed the literature review (study screening and study selection). IG and TT performed the data extraction from the studies included in the review. RC, FS, AMa, AL, SS, MC, and AMi analyzed and interpreted the data extracted from the studies included in the review. RC and FS wrote the first draft of the manuscript. All authors contributed to manuscript revision, read and approved the submitted version.

### Conflict of Interest Statement

The authors declare that the research was conducted in the absence of any commercial or financial relationships that could be construed as a potential conflict of interest. The handling Editor declared past co-authorships with several of the authors RC, FS, AMa, TT, SS, MC, and AMi.

## Supplementary Material

Supplementary Figure 1 Flow-chart showing the literature search and systematic review process according to the Preferred Reporting Items for Systematic Reviews and Meta-analyses (PRISMA) statement recommendations.

Supplementary Table 1 Risk of bias assessment among studies included in the review according to the Newcastle-Ottawa-Scale (NOS) for non-randomized studies and to the Cochrane risk of bias assessment tool for randomized trials. / = not applicable; Un = unclear. The overall quality of evidence was assessed according to Grading of Recommendations Assessment, Development, and Evaluation (GRADE) recommendations **(53)**.
