# Supplementary figures and images for: Templates of Lymph Node Dissection for Renal Cell Carcinoma: A Systematic Review of the Literature
Source: Front Surg. 2018 Dec 19;5:76. doi: 10.3389/fsurg.2018.00076 (PMC6306033; doi:10.3389/fsurg.2018.00076)

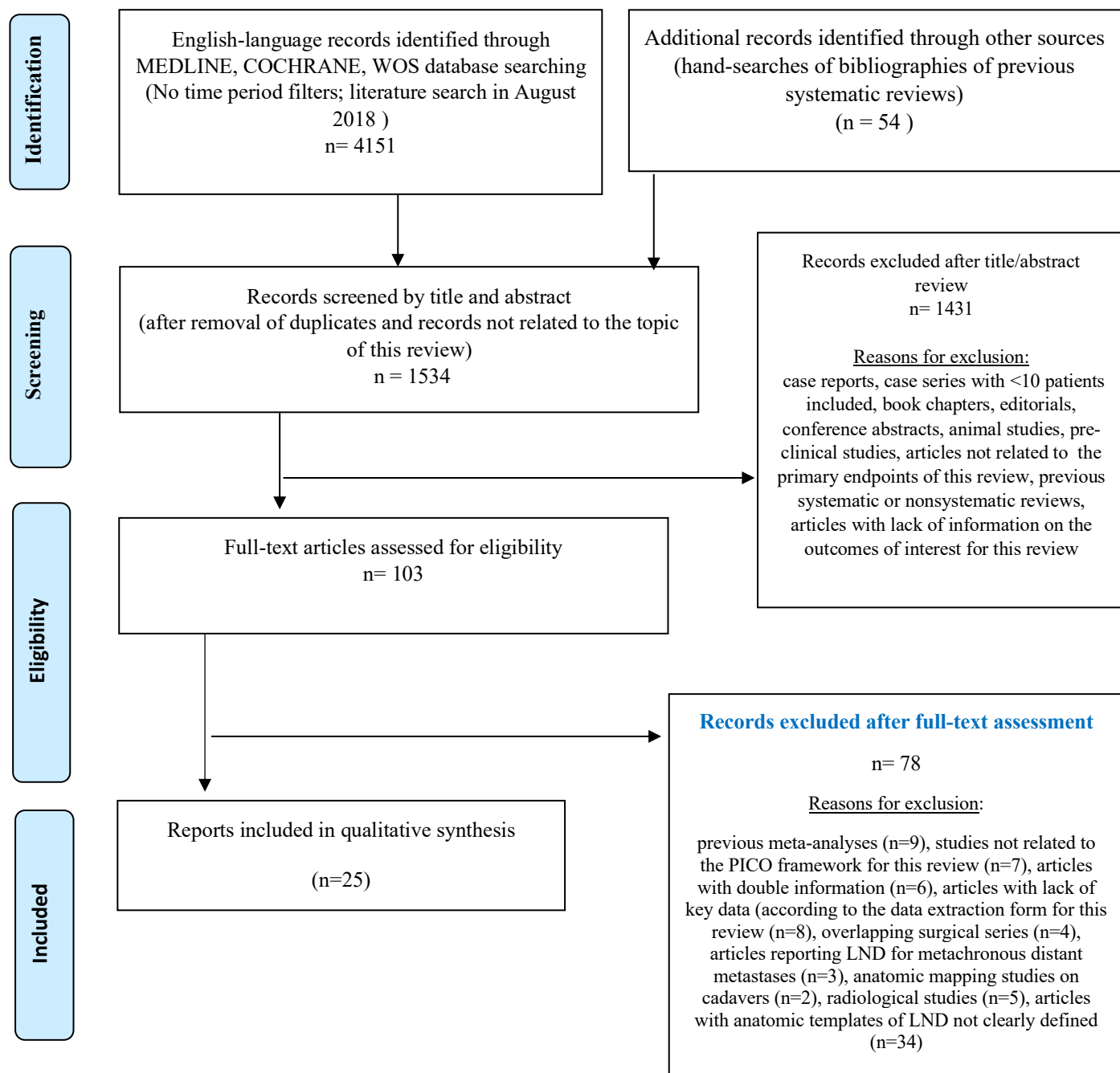

Supplement: Supplementary Figure 1 — Flow-chart showing the literature search and systematic review process according to the Preferred Reporting Items for Systematic Reviews and Meta-analyses (PRISMA) statement recommendations. [file Image_1.pdf]
