# Supplementary material for: Templates of Lymph Node Dissection for Renal Cell Carcinoma: A Systematic Review of the Literature
Source: Front Surg. 2018 Dec 19;5:76. doi: 10.3389/fsurg.2018.00076 (PMC6306033; doi:10.3389/fsurg.2018.00076)
Supplement: Supplementary Table 1 — Risk of bias assessment among studies included in the review according to the Newcastle-Ottawa-Scale (NOS) for non-randomized studies and to the Cochrane risk of bias assessment tool for randomized trials. / = not applicable; Un = unclear. The overall quality of evidence was assessed according to Grading of Recommendations Assessment, Development, and Evaluation (GRADE) recommendations (53). [file Table_1.docx]

| **Retrospective/Prospective Study**  (Authors, Year, Citation) | **Newcastle-Ottawa Scale (NOS)** | | | | | | | | |
| --- | --- | --- | --- | --- | --- | --- | --- | --- | --- |
|  | **Selection (S)** | | | | **Comparability (C)** | **Outcome (O)** | | | **Overall assessment** |
|  | **S.1.** | **S.2.** | **S.3.** | **S.4.** | **C.**  *(cT stage, cN stage)* | **O.1.** | **O.2.** | **O.3.** |  |
| Siminovitch et al 1982 (51) | / | / | * | * | / | / | * | * | **/-/** |
| Giuliani et al 1990 (29) | / | / | * | * | / | / | * | * | **/-/** |
| Herlinger et al 1991 (35) | / | / | * | * | / | / | * | * | **/-/** |
| Minervini et al 2001 (36) | / | / | * | * | * | / | * | * | **/*/** |
| Terrone et al 2003 (25) | * | / | * | * | / | * | * | * | ***/-/** |
| Terrone et al 2006 (26) | * | / | * | * | / | / | * | * | ***/-/** |
| Simmons et al 2007 (20) | / | / | * | * | / | / | * | * | **/-/** |
| Chapman et al 2008 (21) | / | * | * | * | * | / | * | * | ***/*/** |
| Capitanio et al 2009 (52) | * | / | * | * | / | * | * | * | ***/-/*** |
| Ming et al 2009 (37) | / | / | * | * | / | / | * | * | **/-/** |
| Crispen et al. 2010 (27) | / | / | * | * | / | * | * | * | **/-/*** |
| Abaza et al 2011 (24) | / | / | * | * | / | / | * | * | **/-/** |
| Bex et al 2011 (18) | / | / | * | * | / | / | * | * | **/-/** |
| Delacroix et 2011 (30) | / | / | * | * | / | / | * | * | **/-** |
| Know et al 2011 (31) | * | / | * | * | / | * | * | * | ***/-/*** |
| Capitanio et al 2012 (32) | * | * | * | * | / | * | * | * | ****/-/*** |
| Mehta et al 2013 (22) | / | * | * | * | ** | / | * | * | ***/**/** |
| Capitanio et al 2014 (10) | * | / | * | * | / | * | * | * | ***/-/*** |
| Feurstein et al 2014 (8) | / | * | * | * | * | / | * | * | ***/*/** |
| Feurstein et al 2014 (23) | / | * | * | * | * | / | * | * | ***/*/** |
| Babaian et al 2015 (33) | / | / | * | * | / | * | * | * | **/-/*** |
| Kuusk et al 2017 (19) | / | / | * | * | / | * | * | * | **/-/*** |
| Dell’Oglio et al 2017 (34) | * | / | * | * | / | / | * | * | ***/-/** |
| Nini et al 2018 (28) | * | / | * | * | / | / | * | * | ***/-/** |

|  |  |  |  |  |  |  |  |  | |
| --- | --- | --- | --- | --- | --- | --- | --- | --- | --- |
| **Randomized controlled trials**  (Authors, Year, Citation) | **Selection bias** | | | **Performance bias** | **Detection bias** | **Attrition bias** | **Reporting bias** | |  |
|  | *random sequence generation* | *allocation concealment* | *allocation after assessment of eligibility and will to participate* |  |  |  |  |  |  |
| Blom et al 2009 (15) | Low | Un | Un | High | Low | Low | Un | |  |
